# Comparative glycoproteomics study on the surface of SKOV3 *versus* IOSE80 cell lines

**DOI:** 10.3389/fchem.2022.1010642

**Published:** 2022-11-22

**Authors:** Ying Zhou, Xiaoyu Cai, Linwen Wu, Nengming Lin

**Affiliations:** ^1^ Department of Clinical Pharmacology, Key Laboratory of Clinical Cancer Pharmacology and Toxicology Research of Zhejiang Province, Affiliated Hangzhou First People’s Hospital, Cancer Center, Zhejiang University School of Medicine, Hangzhou, Zhejiang, China; ^2^ Department of Clinical Pharmacy, Key Laboratory of Clinical Cancer Pharmacology and Toxicology Research of Zhejiang Province, Affiliated Hangzhou First People’s Hospital, Zhejiang University School of Medicine, Hangzhou, Zhejiang, China

**Keywords:** differential N-glycosylation, site-specific, structure-specific, quantitative N-glycoproteomics, GPSeeker

## Abstract

**Objective:** Site**-** and structure-specific quantitative N-glycoproteomics study of differential cell-surface N-glycosylation of ovarian cancer SKOV3 cells with the non-cancerous ovarian epithelial IOSE80 cells as the control.

**Methods:** C18-RPLC-MS/MS (HCD with stepped normalized collision energies) was used to analyze the 1: 1 mixture of labeled intact N-glycopeptides from SKOV3 and IOSE80 cells, and the site- and structure-specific intact N-glycopeptide search engine GPSeeker was used to conduct qualitative and quantitative search on the obtained raw datasets.

**Results:** With the control of the spectrum-level false discovery rate ≤1%, 13,822 glycopeptide spectral matches coming from 2,918 N-glycoproteins with comprehensive N-glycosite and N-glycan structure information were identified; 3,733 N-glycosites and 3,754 N-glycan sequence structures were confirmed by site-determining and structure-diagnostic fragment ions, respectively. With the control of no less than two observations among the three technical replicates, fold change ≥1.5, and *p*-value ≤ 0.05, 746 DEPGs in SKOV3 cells relative to IOSE80 cells were quantified, where 421 were upregulated and 325 downregulated.

**Conclusion:** Differential cell-surface N-glycosylation of ovarian cancer SKOV3 cells were quantitatively analyzed by isotopic labeling and site- and structure-specific N-glycoproteomics. This discovery study provides putative N-glycoprotein biomarker candidates for future validation study using multiple reaction monitoring and biochemical methods.

## Introduction

Up to now, glycosylation is considered one of the most abundant post-translational modifications (PTMs) found in mammalian cells (Varki and Freeze, 1994). More and more studies have confirmed that glycan structures attached to lipids and proteins play an important role in regulating various physiological and biological events (Agard and Bertozzi, 2009; Kiessling and Splain, 2010). Generally, glycosylation affects cell functions, including cell movement, inflammation, cell–cell adhesion, signal transduction, and virus entry (Nguyen et al., 2017). Meanwhile, a tremendous amount of research has implied that alterations to these processes by aberrant glycan expression could be easily linked to oncogenic transformation through regulating tumorigenesis, tumor cell invasion, and metastasis in various cancers (RodrIguez et al., 2018; Reily et al., 2019; Greville et al., 2020) and influencing some other fundamental processes such as stem cell differentiation (Amano et al., 2010) and embryogenesis (Haltiwanger and Lowe, 2004).

Ovarian cancer has been causing more and more deaths in recent years and remains the second deadliest gynecologic cancer worldwide despite tremendous advances that have been made in diagnostics and treatments over the last few decades (Lheureux et al., 2019). Due to ovarian cancer having high degrees of variability, high proportions of chemo-resistance, and high-relapse rates, most patients relapse only within 3 years after diagnosis (Buechel et al., 2019). Therefore, it is urgently required to find highly specific and sensitive diagnostic biomarkers capable of detecting the disease early. In 1964, abnormal glycosylation was found for the first time by periodic acid Schiff staining on mucin polysaccharide in ovarian cancer tissue sections (Garcia-Bunuel and Monis, 1964). Over the following decades, the involvement of glycosylation in ovarian cancer has then generously proved both in *in vivo* and *in vitro* settings (Abbott et al., 2010). For example, in 1979, Gehrke et al. revealed that quantitative changes in fucose, galactose, and mannose levels determined by gas–liquid chromatography could distinguish the ovarian cancer patient’s serum (Gehrke et al., 1979). Similarly, in 1979, Chatterjee and Barlow et al. found that sialyl, galactosyl, and fucosyltransferase activities were remarkably increased in tissues from ovarian cancer relative to normal tissues (Chatterjee et al., 1979). The results of the study in 1988 using chromatography with a combination of HPLC analysis suggested that levels of glycosylation on purified alpha-1 anti-trypsin differ in the sera from healthy and ovarian cancer patients (Goodarzi and Turner, 1995). These results make it clear that the emergence of glycosylation is of great significance for ovarian cancer; the glycoproteomics measurements are becoming far more common in identifying the key factors in ovarian cancer.

With the advances in the mass spectrometry technique, identifying specific glycosyl biomarkers and seeking strategies for specific glycan epitopes have become a widely pursued research direction (Saldova et al., 2007). In the past years, site-specific N-glycoproteomics has been developed rapidly, providing information on N-glycosites, peptide backbone amino acid sequences, and N-glycan moiety monosaccharide composition. Several bioinformatics tools such as Glycopeptide Search (Chandler et al., 2013), GPFinder 3.0 (Strum et al., 2013), I-GPA (Park et al., 2016), pGlyco 2.0 (Liu et al., 2017), and Mascot (Bollineni et al., 2018) are also used to characterize N-glycans with different expressions in large scale at a composition level in various cancers. However, quantitation of the N-glycans at structural and functional levels and more integrated bioinformatic databases are still urgently needed.

In this study, we combined the C18-RPLC-MS/MS (HCD) analysis with the N-glycan database engine GPSeeker search (Xu et al., 2020) and compared the characteristics of differentially expressed glycopeptide spectral matches (DEGSMs) in serous ovarian cancer SKOV3 cell lines, the most frequent histotype among all epithelial ovarian cancers, relative to non-cancerous ovarian epithelial IOSE80 cell lines. GPSeeker adopts a human theoretical N-glycan database with a monosaccharide sequence and linkage information built from known biological rules and the retrosynthetic strategy (Xiao and Tian, 2019a; Yang et al., 2020). In addition to the spectrum-level false discovery rate (FDR) control using the target-decoy search scheme, N-glycosites are further localized with G-bracket, which is defined as the number of N-acetylglucosamine-containing site-determining peptide fragment ion pairs, each of which can independently confine the N-glycosite; sequence structures are further confirmed with the glycoform score (GF score), which is defined as the number of structure-diagnostic fragment ions, each of which can unambiguously differentiate the structure from other sequence structures with the same molecule formula or monosaccharide compositions. A total of 13,822 glycopeptide spectral matches matching 3,733 peptides, 3,754 N-glycosites, and 2,918 N-glycoproteins were qualitatively authenticated. In addition, 746 DEGSMs were extracted in SKOV3 cells relative to IOSE80 cells, among which, 421 was up-regulated and 325 was down-regulated. Our study provided new insight to unravel the functional role of N-glycosylation in ovarian cancer cells.

## Materials and methods

### Reagents and chemicals

The Roswell Park Memorial Institute (RPMI) 1640 medium and fetal bovine serum (FBS) were purchased from Gibco (Grand Island, NY, United States). The BCA assay kit was purchased from Sangon Biotech (Shanghai, China). Dithiothreitol (DTT, 3483–12–3), 2,2,2-trifluoroethanol (TFE, 99%, 75–89–8), acetaldehyde-^13^C2 (^13^CH_3_^13^CHO, 99 atom % 13C, 1632–98–0), acetaldehyde (CH_3_CHO, 99%, 75–07–0), sodium cyanoborohydride (NaBH_3_CN, 25,895–60–7), ammonia solution (NH_4_OH, 7664–41–7), acetonitrile (ACN, 75–05–8), formic acid (FA, 64–18–6), trypsin, and trifluoroacetic acid (TFA) were purchased from Sigma-Aldrich (St. Louis, MO, United States). Ultra-pure water was obtained on-site using the Millipore Simplicity System (Billerica, MA, United States). Ammonium bicarbonate (ABC) was purchased from Sangon Biotechnology (Shanghai, China). ZIC-HILIC particles were purchased from Thermo Fisher Scientific (Waltham, MA United States). PNGase F was purchased from New England Biolabs (Ipswich, MA, United States).

### Cell culture

Human SKOV3 and IOSE80 cells were obtained from the cell bank of the Chinese Academy of Sciences (Shanghai, China). All cells were cultured in the RPMI 1640 medium supplemented with 10% FBS and incubated at 37°C in a humidified atmosphere with 5% CO_2_ confluence.

### Protein extraction

Human SKOV3 and IOSE80 cells were harvested at about 90% confluence. The cells were washed with cold phosphate-buffered saline, then scraped off cell culture plates using lysis buffer containing 0.1M Tris-HCL, 4% SDS, protease, and phosphatase inhibitors on ice, and were sonicated for 30 min, respectively. The cell debris was then dislodged by centrifugation for 15 min at 14000 g. Protein concentration was measured by BCA assay and adjusted to 1 mg/ml for −80°C storage.

### Reduction and alkylation

The protein concentrations collected previously from both SKOV3 and IOSE80 cells were first reduced in 10 mM DTT at 55°C for 30 min. Thereafter, 100 μl of 200 mM IAA was used for alkylation in the dark for 30 min. Finally, at room temperature (RT), 10 mM DTT was used to quench the alkylation reaction for 30 min.

### Digestion, desalting, and ZIC-HILIC enrichment

The obtained protein concentrate was added with trypsin, according to the ratio of protein: the enzyme is 1:50 (w/w) at the condition of 37°C overnight for digestion. At the end of the reaction, the enzymatic digestion solution was evaporated.

The digested peptides were then desalted by using a C18 (Phenomenex, 15 μm, 300 Å) SPE column at the ratio of protein: packing was 1:50 w/w. Then, before vacuum evaporation (SpeedVac), gradient elution was carried out from 400 μL 50% ACN (0.1% TFA) to 400 μL 80% ACN (0.1% TFA).

For zwitterionic hydrophilic interaction chromatography (ZIC-HILIC) enrichment, the concentrates were then redissolved in 80% ACN with 5% TFA, and the final concentration was adjusted to 5 μg/μl. The re-dissolved solution was then packed into a column of pro-ZIC particles (Merck Millipore, 5 μm, 300 Å) at the ratio of intact glycopeptides to particles of 1:30 (w/w) and then enriched. After being bound, the enriched N-glycopeptide was subsequently eluted by 300 μl 0.1% TFA three times, followed by 200 μl 50 mM NH_4_HCO_3_. All the combined eluents were then dried in the SpeedVac evaporator.

### Isotopic labeling

Isotopic labeling of sugar chains was prepared as described previously (Xiao and Tian, 2019b). In brief, the enriched dry N-glycopeptides from SKOV3 and IOSE80 cells were redissolved in 100 μl TFE. Then, the solutions were mixed with 20% CH_3_CHO (light) or 20% ^13^CH_3_^13^CHO (heavy) at the ratio of 0.25 μl/μg. After immediately vortexing for 1 min, the same volume of 0.6M NaBH_3_CN was added and oscillated at 37°C for 1 h. Finally, the same volume of 4% NH_4_OH was used to terminate the reaction. Heavy-labeled and light-labeled N-glycopeptides were mixed at the ratio of 1: 1, and then dried and stored in the vacuum. Finally, the pellets were resuspended in 50 μL H_2_O for further study.

### C18-RPLC-MS/MS (HCD) analysis

Before C18-RPLC-MS/MS (HCD) analysis, a repeated desalination step was performed on the enriched and labeled N-glycopeptides. Thereafter, the mixture of intact SKOV3 and IOSE80 N-glycopeptides was dissolved in 30 μ L ultra-pure water, separated into three technical replicas (TRs: TR1, TR2, and TR3), and processed by C18-RPLC on the Dionex Ultimate 3000 RSLCnano HPLC system (Thermo Fisher Scientific, San Jose, CA, United States). The separated N-glycopeptides samples were trapped on the 5-cm column (360 μod × 200 μid) and were separated on the 75-cm analytical column (360 μod × 75 μid). Both columns were filled with Phenomenex Jupiter C18 particles (5 μm, 300 Å). The mobile phase was buffer A: mixture of 99.9% H_2_O and 0.1% FA; and buffer B: mixture of 99.9% ACN and 0.1% FA. The loading flow rate for the mobile phase was set constantly at 5 μL/min, and the separation flow rate for the mobile phase was set at 30 nL/min. A sequential procedure of the multi-step gradient elution was set up: 2% buffer B within 12 min for loading samples; 2%–40% buffer B increased in 188 min for elution; increased to 95% buffer B within the following 10 min and held for another 5 min for elution; and decreased to 2% buffer B in the last 25 min for equilibration.

To further detect eluted N-glycopeptides, a Q Exactive mass spectrometer with nano-ESI tandem (Thermo Fisher Scientific, San Jose, CA, United States) was applied: spray voltage at 1.9 kV and capillary tubes at 300°C.

To acquire MS spectra, the following settings were used on the Q Exactive Orbitrap MS: m/z range at 700–2000, automatic gain control target at 3×10^5^, mass resolution at 60 k, and maximum injection time at 20 ms.

To acquire MS/MS spectra in the top 20 data-dependent acquisition modes, the following settings were applied: automatic gain control target at 2×10^5^; mass resolution at 30 k; maximum injection time at 250 ms; isolation window at 3.0 m/z; dynamic exclusion at 20.0 s; and stepped HCD normalized collision energies at 20%, 30%, and 31%.

### Identification by GPSeeker

GPSeeker then analyzed the PRLC-MS/MS (HCD) raw datasets (TR1, TR2, and TR3) for intact N-glycopeptide identification and quantitation. Details were recorded previously (Xiao and Tian, 2019a; Xiao and Tian, 2019b). Briefly, in order to search the SKOV3 and IOSE80 cell intact N-glycopeptides, four theoretical databases were edited (two labels: light and heavy diethylation (L/H) and two directions: forward and reverse (F/R)) based on the human proteome pre-downloaded from UniProt (20,375 entries, http://www.uniprot.org/) and putative human N-glycan databases in GPSeeker. To customize the initial target and decoy searches, protease was set trypsin, the missed cleavage number was allowed to be 1, and N-acetylglucosylation was selected and added as dynamic PTMs. Static PTMs adopted in this research included light [(^12^CH_2_^12^CHO)_2_] and heavy [(^13^CH_2_^13^CHO)_2_] demethylation on the N-terminus and lysine, and reductive alkylation on cysteine and were designated the m/z measuring range of 700–2000. To set the search parameters in the MS spectra, the isotopic peak m/z deviation (IPMD) was set at 20.0 ppm, the isotopic peak abundance cutoff (IPACO) at 40%, and isotopic peak abundance deviation (IPAD) at 50%. Refinement of parameters to obtain further N-glycopeptide spectrum matches (GPSMs) was performed as follows: the top four abundant Y1 ions were set to MS/MS spectra, and the minimum percentage of peptide matching products ions was set to ≥10%. Each pair of the three technical replicate was searched, and the target and decoy GPSMs were combined in a MS excel. After being ranked with decreasing pg-scores, a critical pg-score was chosen to ensure FDR ≤1%. Qualitative information, including the N-glycan identifications (IDs), structure-diagnostic ions, N-glycosites, composition and linkage of N-glycans, and corresponding N-glycoprotein accession numbers, was finally obtained for further study after removing the duplicates.

### Relative quantitation by GPSeekerQuan

Based on the matching results from GPSeeker, we then used the GPSeekerQuan tool to find out the precursor ions in the first-order mass spectrum corresponding to each ID. For each precursor ion isotope profile, a sum of the peak intensity of the first three isotopes was collected for relative quantification, and the relative ratio of the IOSE80/SKOV3 group was calculated. To further screen the final differentially expressed N-glycopeptides IDs, quantitative results showed that at least two among the three TRs were required to be observed. In addition, FC should be controlled at ≥1.5, and the *p*-value calculated by the *t*-test should be classified as < 0.05.

## Results

**FIGURE 1:**
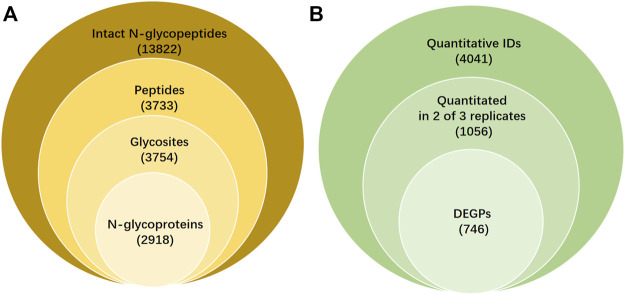
Qualitative and quantitative results and differentially expressed intact N-glycopeptides (DEPGs) in SKOV3 and IOSE80 cells. **(A)** Qualitative information on intact N-glycopeptides IDs including glycosites, peptides, and N-glycoprotein identified by GPSeeker with FDR ≤1% from C18-RPLC-MS/MS (HCD) analysis in 1:1 mixed SKOV3 and IOSE80 cells. **(B)** Quantitative results of differentially expressed N-glycopeptides searched by GPSeekerQuan.

Both SKOV3 and IOSE80 cells were treated as described in the section “Material and Methods”. After ZIC-HILIC enrichment and isotopic labeling, the intact N-glycopeptide was blended at the ratio of 1:1 and used for C18-RPLC-nanoESI-MS/MS (HCD) online analysis. Three TRs (TR1, TR2, and TR3) were obtained. All the qualitative information on intact N-glycopeptide IDs, N-glycosylation sites, characteristic peptides, and intact N-glycoproteins was identified by GPSeeker, a newly developed N-glycopeptide search engine, which possesses the condition of removing duplicates and controls the FDR ≤1%. A total of 13,822 glycopeptide spectral matches (Supplementary Table S1) were identified corresponding to 3,733 unique peptides (Supplementary Table S2), 3754 N-glycosites (Supplementary Table S3), and 2918 N-glycoproteins (Supplementary Table S4) from the TRs (Figure 1A).

Among the 13,822 N-glycopeptide IDs, 6,659 was appraised the GF score ≥1. The GF score was set as the standard numerically as the number of structural diagnostic ions in the matching fragment ions for each ID. For each of the 13,822 N-glycopeptide IDs, detailed information including the spectrum index, retention time (RT, min), the m/z isolation window of precursor ions (Iso.m/z), experimental m/z (Exp.m/z), theoretical m/z (Theo.m/z), IPMD (ppm), accession number with the corresponding protein ID, protein name, structure composition, N-glycan linkages (g-linkage), N-glycan sites (GlycoSite), -log (*p* score), glyco-bracket (Gly-bracket), and GF scores were made up as a list in Supplementary Table S1 **.** Statistics on the putative g-linkages, complex, hybrid, and high-mannose percentages were 77.74%, 16.94% and 5.32%.

**FIGURE 2:**
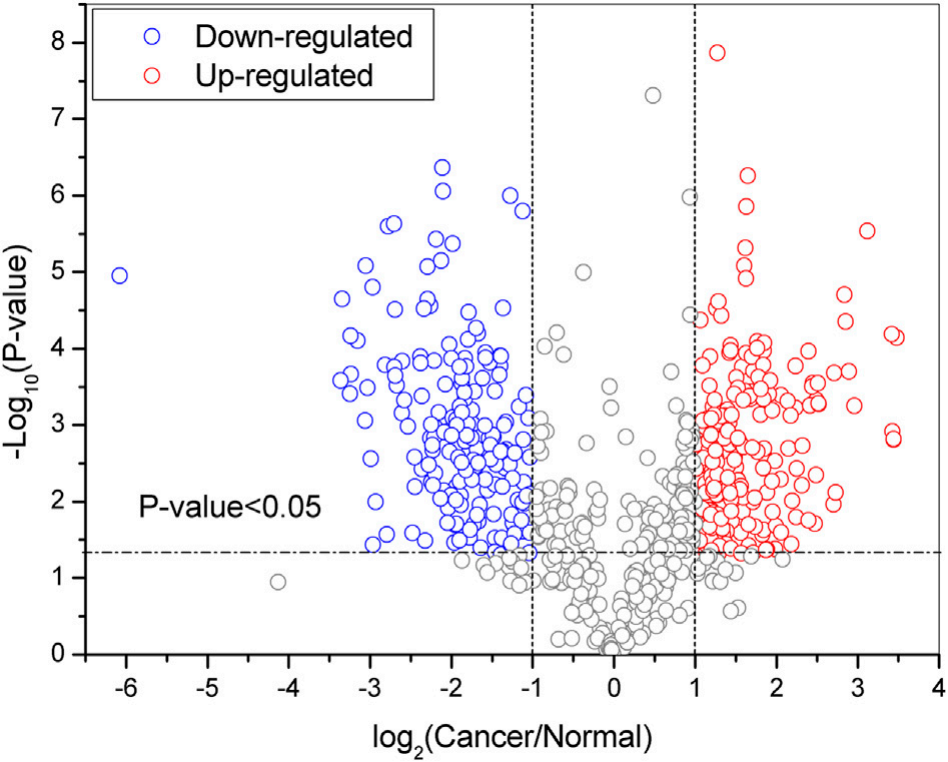
Volcano plot of DEPGs (at least two technical duplications three times) in SKOV3 vs. IOSE80 cells. Red: upregulation. Blue: downregulation. *p* < 0.05.

Based on the quantitative results from GPSeeker, the precursor ions in the first-order mass spectra corresponding to each ID were then sought by the GPSeekerQuan tool. With the standard of screening the three most ample isotopic peaks, 4041 IDs were quantified **(** Supplementary Table S4 **).** Among them, 1056 IDs were quantified at least twice from the three TRs (Supplementary Table S5). By setting the filter range further with FC ≥ 2 and *p* < 0.05, 746 DEGSMs were identified (Figure 1B, Supplementary Table S6-7), where 421 DEGSMs were upregulated, and 325 DEGSMs were downregulated (Figure 2).

**FIGURE 3:**
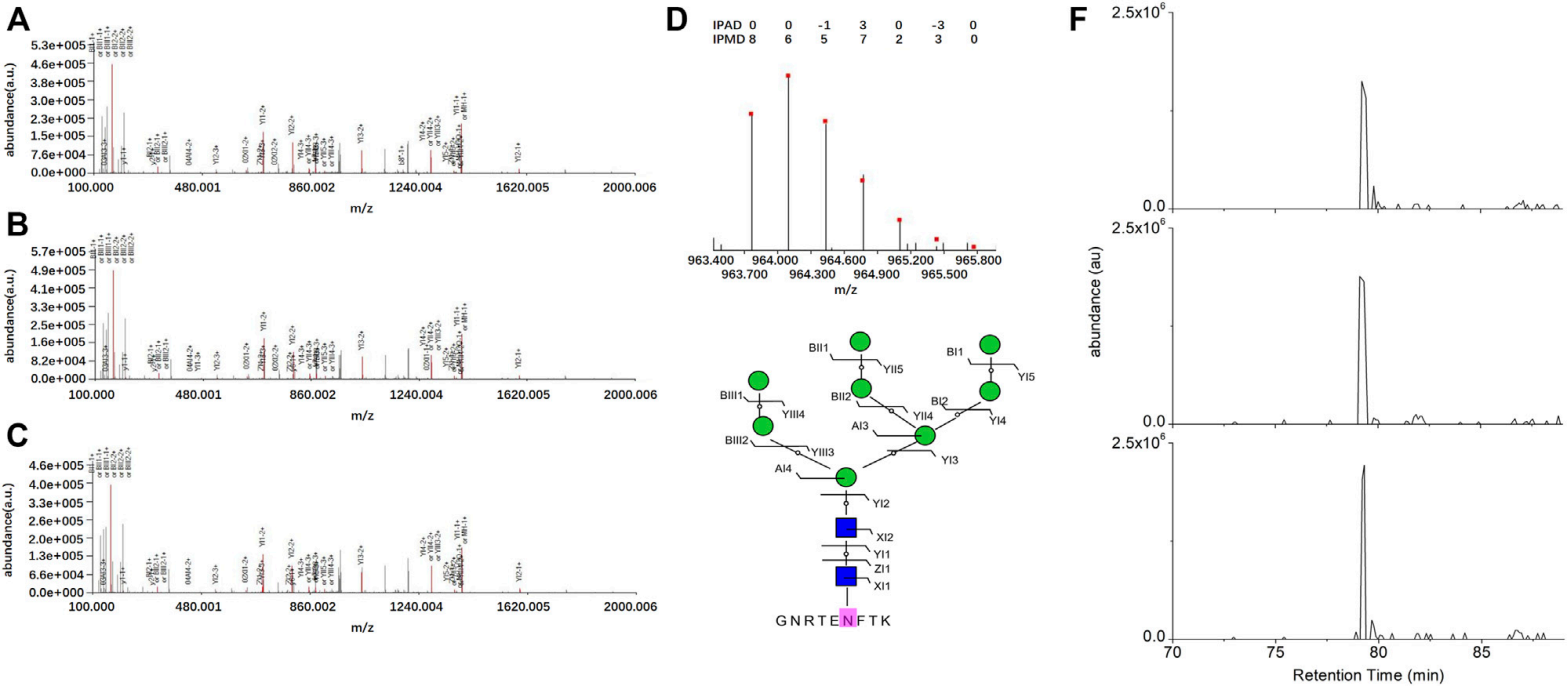
Identification of the intact N-glycopeptide GNRTENFTK_N2H8. **(A–C)** Annotated MS/MS spectra from the three technical replicate RPLC-MS/MS runs; **(D)** fingerprinting map of the experimental (bar) and theoretical (dot) isotopic envelopes of the precursor ion; **(E)** graphical fragmentation map of the N-glycan moiety; and **(F)** extracted ion chromatograms of the precursor ion from the three technical replicate RPLC-MS/MS runs.
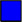
= N-acetylglucosamine (Y),
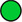
= mannose (M); au = arbitrary unit; IPMD = isotopic peak m/z deviation; IPAD = isotopic peak abundance deviation.

For instance, the intact N-glycopeptide GNRTENFTK_N2H8F0S0 of the N-glycosite on N564 from mitochondrial N-glycoprotein Poly(A) RNA polymerase(PAPD_HUMAN, Q9NVV4) displayed the highest upregulation in SKOV3 cells relative to IOSE80 cells (Figure 3).

**FIGURE 4:**
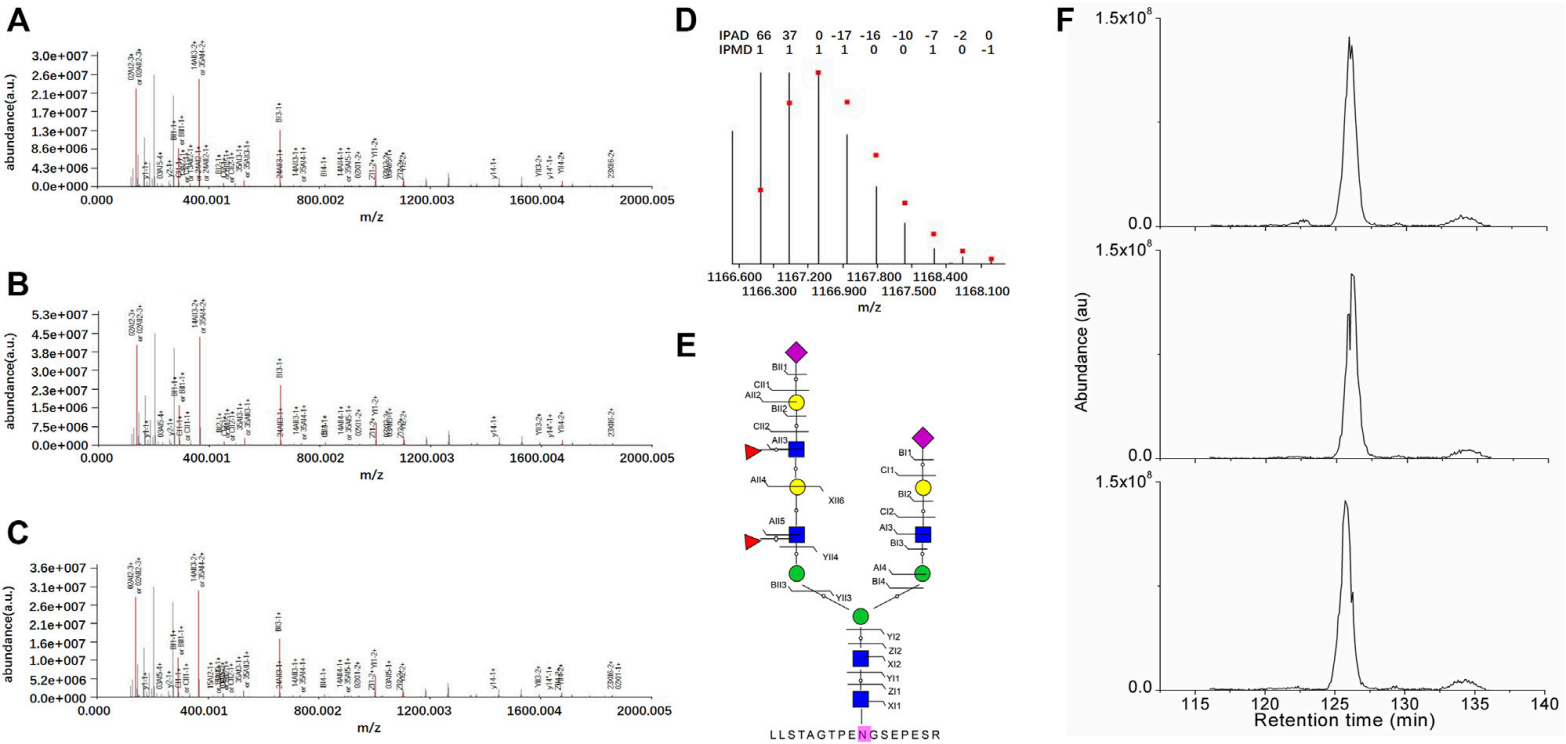
Identification of the intact N-glycopeptide LLSTAGTPENGSEPESR_N5H6F2S2. **(A–C)** Annotated MS/MS spectra from the three technical replicate RPLC-MS/MS runs; **(D)** fingerprinting map of the experimental (bar) and theoretical (dot) isotopic envelopes of the precursor ion; **(E)** graphical fragmentation map of the N-glycan moiety; and **(F)** extracted ion chromatograms of the precursor ion from the three technical replicate RPLC-MS/MS runs.
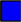
=N-acetylglucosamine (Y);
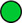
=mannose (M);
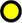
= galactose (L);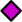 =sialic acid (S);
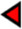
= fucose **(F)**; au = arbitrary unit; IPMD = isotopic peak m/z deviation; IPAD = isotopic peak abundance deviation.

The intact N-glycopeptide LLSTAGTPENGSEPESR_ N5H6F2S2 from the N-glycosite on N621 of N-glycoprotein Polycystin-1 (PKD1_HUMAN, P98161) displayed the lowest downregulation in SKOV3 cells relative to IOSE80 cells (Figure 4).

**FIGURE 5:**
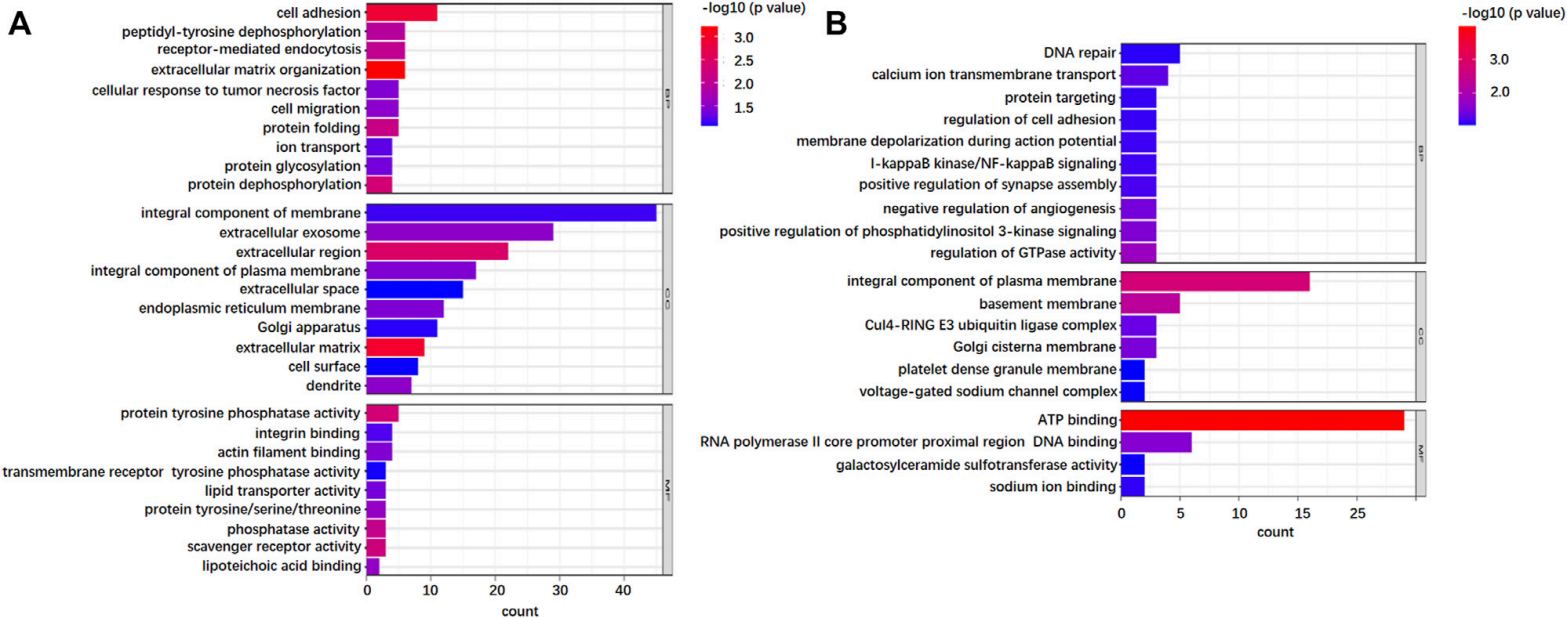
Gene Ontology (GO) analysis of the identified N-glycoproteins from 736 DEPGs. **(A)** GO enrichment of top 10 biological process (BP), cellular component (CC), and molecular function (MF) terms for 421 upregulations. **(B)** GO enrichment of top 10 B P, CC, and MF terms for 325 downregulations.

In order to further understand the potential functional mechanism of 746 DEGSMs and corresponding genes, we performed Gene Ontology (GO) and the Kyoto Encyclopedia of Genes and Genomes (KEGG) enrichment analysis by DAVID Bioinformatics Resources 6.8 (https://david.ncifcrf.gov/). All terms are enriched and divided into three functional groups: biological processes (BPs), cellular components (CCs), and molecular functions (MFs) (Figure 5).

**FIGURE 6:**
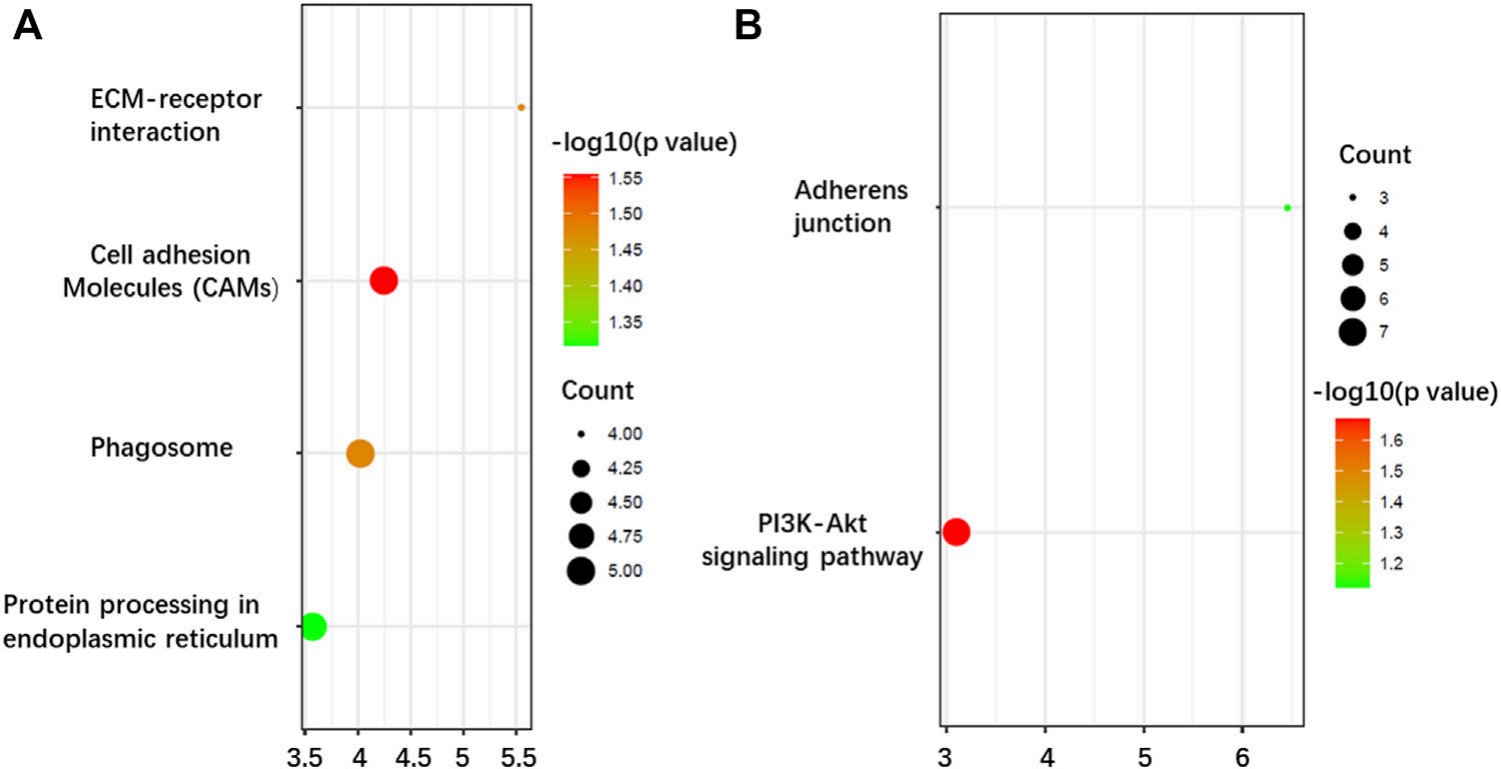
Kyoto Encyclopedia of Genes and Genomes (KEEG) pathway enrichment analysis of the N-glycoproteins corresponding to the DEPGs in SKOV3 relative to IOSE80 cells. **(A)** Enrichment of 421 upregulated genes. **(B)** Enrichment of 325 downregulated genes.

As far as BP is concerned, both upregulation and downregulation were active in the cell adhesion process. However, upregulation was more involved in cell adhesion, while downregulation was more active in DNA repair. For CC analysis, both up- and downregulation were circulated mainly in integral components of the membrane. Furthermore, upregulations also circulated in the extracellular region, which implied that after complete glycosylation modification, proteins were effectively secreted to the extracellular domain and exercised effective biological regulatory functions. The downregulations showed mostly membrane circulation without the extracellular region. In addition, some of the downregulations function specifically as the components of the Clu4-RING E3 ubiquitin ligase complex and voltage-gated sodium channel complex. The results of MF showed that upregulations exhibited significant enrichment in the activities of protein tyrosine phosphatase activity, integrin, and actin filament binding, while downregulations exhibited significant enrichment in the activities of ATP-binding. KEGG pathway analysis further indicated that the upregulations were enriched in cell adhesion molecules, phagosome, ECM-receptor interaction, and protein processing in the endoplasmic reticulum (Figure 6A), while downregulations were focused on only two pathways: PI3K-Akt signaling pathway and adherens junctions (Figure 6B).

## Discussion

Ovarian cancer is one of the leading causes of gynecological mortality (Beg et al., 2022). This disease is usually diagnosed late and lacks an effective screening strategy (Matulonis et al., 2016). In addition, about 90% patients relapse and eventually develop drug resistance (Agarwal and Kaye, 2003). Therefore, it is an urgent need to identify specific biomarkers of this disease so as to conduct early detection and find new therapeutic targets. An important metastatic feature of epithelial ovarian cancer is that after detachment of malignant cells from the primary site into the peritoneal fluid, they form multicellular aggregates with enhanced survival mechanisms in a suspended state and re-adhere to the surface of the peritoneum to form secondary tumors (Dhaliwal and Shepherd, 2022). Therefore, the ability of cells to contact each other and to adhere to the peritoneum when ovarian cancer cells are suspended is critical for metastasis of ovarian cancer cells. Extensive metastasis is one of the key factors leading to the death of ovarian cancer (Bilbao et al., 2021). Our research found that multiple glycoproteins associated with cell adhesion were differentially expressed in ovarian epithelial cancer SKOV3 cells vs. non-cancerous ovarian epithelial IOSE80 cells, including VTN, VCAM1, ALCAM, CNTN5, PTK7, LAMC1, RGMB, THBS1, DSCAML1, PCDHA6, CD6, and UMOD (Supplementary Table S6-7), and participated in cell adhesion-related signaling pathways. In addition, there are a series of cell adhesion-related mechanisms of DEPGs involved in adhesion (such as protein targeting, ECM–receptor interaction, and adherens junction and cell migration). In view of the important role of cell adhesion in ovarian cancer cells, we selected some representative cell adhesion-related DEGSMs to discuss and forecast its potential targeting effect in the progression of ovarian cancer.

### CD274/PD-L1

Among the identified differential N-glycopeptides, we found that the CD274 molecule (CD274_HUMAN, Q9NZQ7), also known as PD-L1, was N-glycosylated at the Asn219 site (Supplementary Table S7). PD-L1 inhibitors, including the already marketed tislelizumab, nivolumab, and pembrolizumab, have shown significant efficacy in tumors, particularly small cell lung cancer and liver cancer (Liu and Wu, 2020; Wang et al., 2021). The breadth, depth, and persistence of its response are very rare, which has become the focus of tumor immunotherapy research in recent years. At present, the main treatment methods for ovarian cancer are surgery combined with radiotherapy, chemotherapy, and targeted therapy, but the survival rate of patients has not been significantly improved (Coughlan and Testa, 2021). Immunotherapy is a new strategy proposed in recent years to treat ovarian malignant tumors, aiming to enhance the ability of patients’ immune system to recognize and attack tumor cells (Morand et al., 2021). Among them, the research on PD-L1 inhibitors in ovarian cancer, especially advanced ovarian cancer, recurrent ovarian cancer, and platinum-sensitive or platinum-resistant ovarian cancer, has been carried out successively (Mittica et al., 2016; Pujade-Lauraine et al., 2018; Zhang et al., 2022). Our findings provide new evidence for the application of the PD-L1 inhibitor in the treatment of ovarian cancer. In addition, a study suggested that PD-L1 carried polyLacNAc glycans mainly in the Asn219 sequon in breast cancer MDA-MB231 cell lines, and the role of glycans on Asn291 influenced its interaction with PD-L1 (Benicky et al., 2021).

Our study also identified that CD274 was occupied by N-glycans on the N-glycosite Asn291. Intact N-glycopeptides with compositions of N5H6F0S2 were found upregulated in the ovarian cancer SKOV3 cells. It is foreseeable that N-glycosylation at site Asn219 is likely to be a specific marker of the tumor, requiring further discovery and verification investigation in ovarian cancer and never promising as a diagnostic or therapeutic marker in the future.

### ALCAM/CD166

The activated leukocyte cell adhesion molecule (ALCAM_HUMAN, Q13740), also known as CD166, is a single chain type 1 transmembrane glycoprotein, containing 10 potential N-glycosylation sites. As the member of the immunoglobulin superfamily (IgSF), CD166 was reported to participate in activation of T cells, neutrophil migration, inflammation, tumor propagation, and angiogenesis (Darvishi et al., 2020). As a valuable prognostic marker of cancers, ALCAM indicated the clinical stage, grade, and the invasiveness of cancers. In addition, as an adhesion molecule, ALCAM also involved in migratory and adhesive properties, played roles in cancer metastasis and leukocyte homing (Kim et al., 2022). Previous studies suggested that endogenous ALCAM modified mostly by *α* (2,6)-sialylated glycans and recombinant glycosylated ALCAM modified mostly by α(2,3)-sialylated glycans in breast carcinoma cells showed different behaviors when binding to Gal-8 (Fernandez et al., 2016). Sialylation of ALCAM influenced its adhesion to Gal-8-coated surfaces, disrupted the cell adhesion, and thus led to breast cancer growth inhibition (Ferragut et al., 2019). Our study showed that ALCAM was N-glycosylated in ovarian cancer, and there were two N-glycosylation sites at Asn90 and Asn95. However, according to information on the g-Linkage, we found that ALCAM was not regulated by sialylated glycans but contained the core fucosylation sites in its LNLSENYTLSISNAR glycopeptide with the g-Linkage 01Y (61F)41Y41M(31M41Y41L31Y)61M61Y41L and 01Y (61F)41Y41M(31M41Y41L31Y41L)61M61Y up-regulated in ovarian cancer cells (**Supplementary Table S7**). Our previous studies suggested that core fucosylation plays important roles in liver cancer proliferation and migration (Zhou et al., 2017); whether core fucosylation of ALCAM has effect on the ovarian cancer progress could be further explored.

### CD6

The CD6 molecule (CD6_HUMAN, P30203) is a type 1 transmembrane glycoprotein expressed in the cell surface of T cells that binds to ALCAM to function as co-stimulatory molecules and take roles in modulating T-cell proliferation, activation, and trafficking (Oh et al., 2019; Rambaldi et al., 2022). A recent study suggested that the anti-CD6 monoclonal antibody, UMCD6, enhanced killing of cancer cells through effects on NK and CD8^+^ cells (Ruth et al., 2021). The role of CD6 in ovarian cancer has not been reported. Our study suggested that CD6 was N-glycosylated in ovarian cancer cells with two N-glycosylation sites at Asn345 and Asn348.

### VCAM1/CD106

Vascular cell adhesion molecule-1 (VCAM-1_HUMAN, P19320), also known as CD106, is a member of the immunoglobulin (Ig) superfamily of adhesion molecules. It was first identified in 1989 (Kumar et al., 1994). VCAM-1 is not expressed or rarely expressed in normal cells, but it is abnormally highly expressed in many tumor tissues. Previous studies showed that VCAM1 was highly expressed in HCC tissues, and most of them showed strong positive expression. Therefore, VCAM-1 and other adhesion molecules were believed to be significantly correlated with the invasion of HCC and could be used as a marker of tumor invasion (Wu et al., 2022). In addition, in the study of breast cancer, the direct role of VCAM1 in tumor metastasis was also confirmed (Lee et al., 2016). In ovarian cancer, there is a strong correlation between the VCAM1 expression level and ascite volume. Targeting the VLA4/VCAM1 pathway can inhibit macrophage-mediated permeability and effectively control formation of ascites (Zhang et al., 2021). Furthermore, the expression of VCAM1 correlates with poor prognosis in ovarian cancer, especially in the high grade of serous ovarian cancer (Zhang et al., 2021). For VCAM1, intact N-glycopeptides along with the Asn365 glycosite were found to be upregulated in the ovarian cancer SKOV3 cells.

### MUC16, MUC15, and MUC20

Mucins (MUCs) are a group of highly glycosylated macromolecules, which are mainly expressed in mammalian epithelial cells. MUCs contribute to the formation of the mucous barrier (Corfield, 2015). Some of the MUCs are ectopic expressed in cancer cells and participate in the occurrence and development of cancers; thus, they were identified as important biomarkers and promising therapeutic targets in the cancer diagnosis and treatment. Among them, MUC16 is very famous (Lee et al., 2021). MUC16 (CA125) is the largest transmembrane mucin, which is highly glycosylated and reportedly promotes ovarian cancer (Zeimet et al., 1998). It is considered a serum biomarker for ovarian cancer due to its overexpression on the cell surface and divides or sheds into the blood (Bast et al., 1998). In our study, we suggested that mucin 16 (MUC16_HUMAN, Q8WXI7) is glycosylated on Asn110 with compositions of N2H6F0S0, Asn1877, and Asn12570 with compositions of N2H8F0S0. However, there is no significant difference in the regulation of N-glycosylation between the two cell lines.

We suggested mucin 15 (MUC15_HUMAN, Q8N387) on the N-glycosite Asn225 with compositions of N5H6F0S3/N4H5F0S2/N5H6F0S2 and mucin 20 (MUC20_HUMAN, Q8N307) on the N-glycosite Asn616 with compositions of N2H8F0S0; both belong to the MUC family and share the similar structure and functional mechanism to MUC16 and were N-glycosylated and upregulated in the ovarian cancer cell line. MUC15 was reported to participate in cell adhesion to the extracellular matrix in cancers, while MUC20 was reported to promote aggressive phenotypes in the epithelial ovarian cancer. However, the glycosylation differences of MUC15 and MUC20 between the cancer and normal cells were not fully explored. We identified the N-glycosylation of MUC15 and MUC20 in ovarian cancer cells; the potential of the MUC15 and MUC20 as the diagnosis marker remains to be investigated in the future.

There are still two major limitations to this study. First, non-cancerous ovarian epithelial IOSE80 cells are essentially a kind of immortalized cell line, which shares some characteristics similar to cancer and cannot completely simulate the state of normal cells *in vivo*. Second, we did not perform any manual validation of our peptide and cannot confirm that all of the hits are real. Thus, further experiments performed on tissues or serum are needed to support our conclusions.

## Conclusion

In this study, we successfully carried out the quantitative structure- and site-specific N-glycoproteomics based on ovarian cancer cell models (SKOV3 relative to IOSE80) and characterized the DEGSMs. The mixture of N-glycopeptides from both SKOV3 and IOSE80 cell lines was detected by C18-RPLC-MS/MS (HCD) after being digested by trypsin, enriched by ZIC-HILIC, and stably labeled by isotopic diethyl. Combined with DB search using GPSeeker, 13,822 glycopeptide spectral matches along with 3754 N-glycosites, 3733 peptide backbones, and 2918 N-glycoproteins were identified. Out of 13,822 glycopeptide spectral matches, 746 DEGSMs were identified, of which 421 were upregulated and 325 were downregulated. The aforementioned quantitative structure N-glycoproteomics centered on GPSeeker will provide new insights, which helps find a new application in the precise analysis of site- or structure-specific DEGSMs with ovarian cancer physiological and pathological relevance. The results also help reveal the relationship between ovarian cancer and N-glycosylation and provide many new and good hypothetical candidates for future research on biomarkers and mechanisms of ovarian cancers.

## Data Availability

The mass spectrometry proteomics data have been deposited to the ProteomeXchange Consortium *via* the iProX partner repository with the dataset identifier PXD030927.

## Abbreviations

ZIC-HILIC, zwitterionic hydrophilic interaction chromatography; PTM, post-translational modification; DEGSM, differentially expressed glycopeptide spectral match; FDR, false discovery rate; FC, fold change; RPMI, Roswell Park Memorial Institute; FBS, fetal bovine serum; TR, technical replica; IPMD, isotopic peak m/z deviation; IPACO, isotopic peak abundance cutoff; IPAD, isotopic peak abundance deviation; GPSM, N-glycopeptide spectrum match; IDs, identifications; GF score, glycoform score; GO, Gene Ontology; KEGG, Kyoto Encyclopedia of Genes and Genomes; BP, biological process; CC, cellular component; MF, molecular function.
