# Supplementary material for: Comparative glycoproteomics study on the surface of SKOV3 versus IOSE80 cell lines
Source: Front Chem. 2022 Nov 22;10:1010642. doi: 10.3389/fchem.2022.1010642 (PMC9723240; doi:10.3389/fchem.2022.1010642)
Supplement: Supplementary file 4 [file DataSheet1.PDF]

(A)

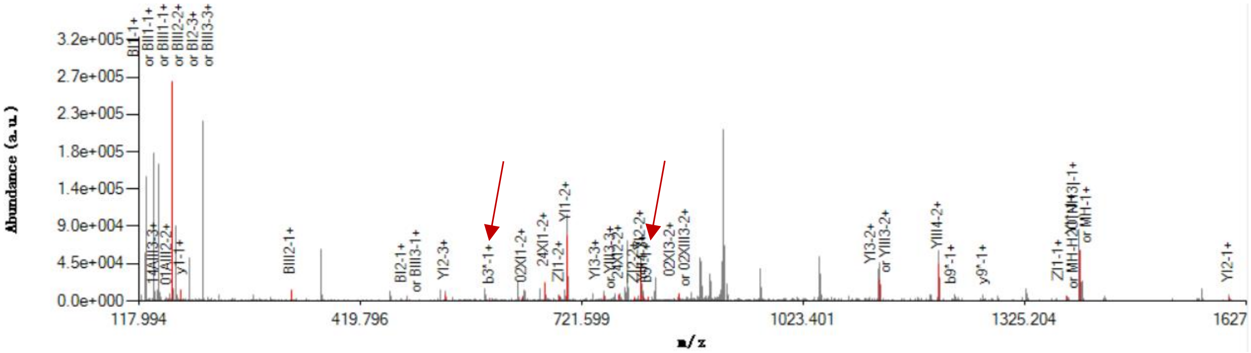


(B)

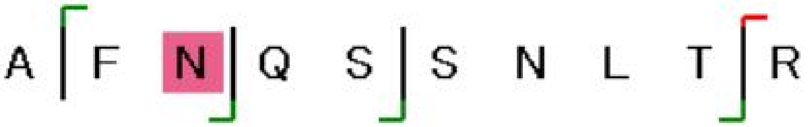


(C)

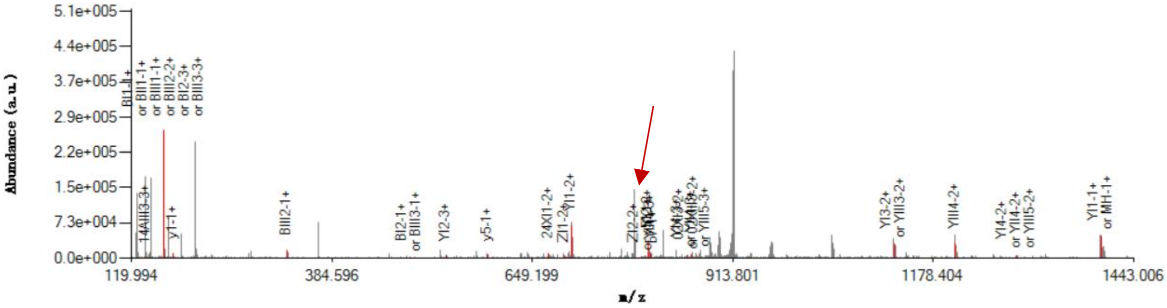


(D)

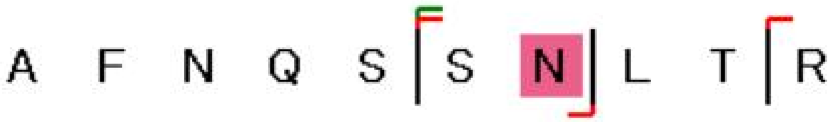


**Supplemental Figure 1.** Identification of N-glycosites N464 and N468 on N-glycoprotein Zinc finger protein 85 (ZNF85_HUMAN, Q03923) with two (**A**, **B**, b3*-1+, b5*-1+) and one (**C**, **D**, y5*-1+) site-determining fragment ions; *=N-acetylglucosamine-containing.
